# Supplementary material for: Contextual barriers and facilitators influencing implementation fidelity of school-based preventive chemotherapy for schistosomiasis: A qualitative study in two endemic districts in the Central Region, Ghana
Source: PLoS One. 2026 Aug 19;21(8):e0348920. doi: 10.1371/journal.pone.0348920 (PMC13489406; doi:10.1371/journal.pone.0348920)
Supplement: Supplementary file 1 [file pone.0348920.s001.docx]

Figure 1: A conceptual framework adopted from the Carroll et al framework showing how the interaction of various factors affects the implementation fidelity of preventive chemotherapy (Carroll et al., 2007).

**Quality of Delivery**

-Availability of drugs

-Availability of tools

-Adherence to dosage

**Intervention Complexity**

-Easiness of delivering

-Geographical nature

-Program setting

**Facilitation Strategies**

-Training and workshop

-Manual and guideline

-Supervision and feedback

-Incentive

**Participant Responsiveness**

-Perception of participants

-Participant participation

-Commitment and readiness

**Community Factors**

-Culture and Religion

-Awareness and Perception

-Community engagement

-Community sensitization

Implementation fidelity

**Individual Factors**

-Attitude and beliefs

-Social and parental influence

-Coverage

-Frequency

-Duration

-Content
