# Supplementary material for: Contextual barriers and facilitators influencing implementation fidelity of school-based preventive chemotherapy for schistosomiasis: A qualitative study in two endemic districts in the Central Region, Ghana
Source: PLoS One. 2026 Aug 19;21(8):e0348920. doi: 10.1371/journal.pone.0348920 (PMC13489406; doi:10.1371/journal.pone.0348920)
Supplement: Supplementary file 2 [file pone.0348920.s002.docx]

**Table 1:** Socio-demographic characteristics of study participants (N = 21)

| **Participant type** | **Number of Participants (N)** | **Sex** | **Age** | **Years of experience** |
| --- | --- | --- | --- | --- |
| **District NTDs Coordinator (KII)** | **4** |  |  |  |
| NTD Program Coordinator 1 |  | Male | 42 | 10 |
| NTD Program Coordinator 2 |  | Male | 46 | 7 |
| NTD Program Coordinator 3 |  | Female | 37 | 6 |
| NTD Program Coordinator 4 |  | Male | 41 | 9 |
| **District SHEP Coordinators (KII)** | **2** |  |  |  |
| SHEP Coordinator 1 |  | Female | 38 | 5 |
| SHEP Coordinator 2 |  | Male | 51 | 13 |
| **SHEP Teachers (IDI)** | **9** |  |  |  |
| SHEP Teacher 1 |  | Female | 28 | 2 |
| SHEP Teacher 2 |  | Female | 39 | 7 |
| SHEP Teacher 3 |  | Male | 35 | 4 |
| SHEP Teacher 4 |  | Female | 27 | 3 |
| SHEP Teacher 5 |  | Male | 43 | 8 |
| SHEP teacher 6 |  | Male | 37 | 5 |
| SHEP Teacher 7 |  | Female | 42 | 8 |
| SHEP Teacher 8 |  | Female | 29 | 4 |
| SHEP Teacher 9 |  | Male | 48 | 11 |
| **Head Teachers (IDI)** | **6** |  |  |  |
| Head Teacher 1 |  | Female | 51 | 12 |
| Head Teacher 2 |  | Male | 49 | 8 |
| Head Teacher 3 |  | Male | 36 | 4 |
| Head Teacher 4 |  | Female | 47 | 10 |
| Head Teacher 5  Head Teacher 6 |  | Female  Female | 44  43 | 6  7 |
| **Total Participants (N)** | **21** |  |  |  |
