# Supplementary material for: Contextual barriers and facilitators influencing implementation fidelity of school-based preventive chemotherapy for schistosomiasis: A qualitative study in two endemic districts in the Central Region, Ghana
Source: PLoS One. 2026 Aug 19;21(8):e0348920. doi: 10.1371/journal.pone.0348920 (PMC13489406; doi:10.1371/journal.pone.0348920)
Supplement: Supplementary file 3 [file pone.0348920.s003.docx]

**Table 2.** Key themes identified as barriers in implementing school-based preventive chemotherapy.

| **Main Themes** | **Sub-Themes** | **CFIR Domain** |
| --- | --- | --- |
| 1. Socio-cultural beliefs and misconceptions on treatment uptake | - Cultural and religious beliefs - Perception and myths surrounding the school-based preventive chemotherapy. - Parental resistance to school-based preventive chemotherapy. | - Outer Setting |
| 2. Drug-related fears and adverse reactions | - Real drug-side effects - Large size of the praziquantel drug - Challenge in managing side effects - Psychosomatic reaction and peer imitation | - Outer Setting |
| 3. Logistical constraints and resource limitations | - Insufficiency of measuring tools - Inadequate transportation logistics | - Inner Setting |
| 4. Lack of training and incentives for frontline implementers | - Inadequate teachers’ training - Lack of incentives for frontline implementers - Shortage of well-trained implementers | - Implementation process |
| 5. Inadequate community sensitization and engagement | - Inadequate pre-MDA community sensitization - Insufficient awareness of the programs | - Implementation process |
| 6. Insufficient program funds | - Inadequate program funding - Delays in funding release - Competing school-based programs | - Inner setting |
| 7. Gaps in Coverage and Fidelity to Protocol | - Low program coverage. - Short duration of program implementation. - Inconsistent frequency of program delivery | - Implementation Process |
