# Supplementary material for: Contextual barriers and facilitators influencing implementation fidelity of school-based preventive chemotherapy for schistosomiasis: A qualitative study in two endemic districts in the Central Region, Ghana
Source: PLoS One. 2026 Aug 19;21(8):e0348920. doi: 10.1371/journal.pone.0348920 (PMC13489406; doi:10.1371/journal.pone.0348920)
Supplement: Supplementary file 4 [file pone.0348920.s004.docx]

**Table 3.** Key themes emerged as facilitators in implementing school-based preventive chemotherapy

| **Themes** | **Sub-themes** | **CFIR Domain** |
| --- | --- | --- |
| 1. Commitment of frontline implementers and role modelling | - Teachers’ commitment to the students’ health - Teachers’ role modelling of swallowing drugs | - Characteristics of Individuals |
| 2. Pre-MDA sensitization/awareness | - Pre-MDA Parent-Teacher Association (PTA) meetings - Community mobilization before MDA. - Engagement of the community in the program | - Implementation Process |
| 3. Collaboration between the health and education sectors. | - Joint implementation between GHS and GES - Collaboration between NTD program coordinators and SHEP coordinators - Support of the Program Coordinator to teachers | - Inner Setting |
