# Supplementary material for: Contextual barriers and facilitators influencing implementation fidelity of school-based preventive chemotherapy for schistosomiasis: A qualitative study in two endemic districts in the Central Region, Ghana
Source: PLoS One. 2026 Aug 19;21(8):e0348920. doi: 10.1371/journal.pone.0348920 (PMC13489406; doi:10.1371/journal.pone.0348920)
Supplement: Supplementary file 5 [file pone.0348920.s005.docx]

**Table 4.** Key Themes Emerged as Recommended Strategies for Improving Implementation Fidelity of School-Based Preventive Chemotherapy for Schistosomiasis.

| **Theme** | **Sub-themes** | **CFIR Domain** |
| --- | --- | --- |
|  |  |  |
| 1. Integrated drug delivery approaches | - Complementing school-based MDA with community-based distribution. - Expanding coverage to out-of-school children. - Strengthening school-based outreach activities. | Outer Setting |
| 2. Technology-assisted monitoring and reporting systems | - Mobile-based reporting applications. - Digital dashboards and electronic tracking systems - GIS-based verification of treatment coverage. | Implementation Process |
| 3. Improved praziquantel formulations | - Development of child-friendly drug formulations with a smaller tablet size for children. - Reduced side effects. And improved palatability. | Intervention Characteristics |
| 4. Enhanced community sensitization and stakeholder engagement | - Early and continuous community sensitization and awareness activities - Strengthening parental engagement through PTA meetings - Involvement of community and religious leaders in sensitization activities | Implementation Process |
