# Supplementary material for: Contextual barriers and facilitators influencing implementation fidelity of school-based preventive chemotherapy for schistosomiasis: A qualitative study in two endemic districts in the Central Region, Ghana
Source: PLoS One. 2026 Aug 19;21(8):e0348920. doi: 10.1371/journal.pone.0348920 (PMC13489406; doi:10.1371/journal.pone.0348920)
Supplement: Supplementary file 6 [file pone.0348920.s006.docx]

**S1 Appendix: In-depth Interview Guide.**

**Section A: Introduction and Demographic Overview**

i. **Introduction and rapport building**

Welcome to this short interview. The purpose of the study is to assess contextual barriers and facilitators influencing implementation fidelity of school-based preventive chemotherapy for schistosomiasis. I would like to ask you a few questions. Your information will be kept confidential, and you have the right to skip any question or withdraw at any time. Please allow me to proceed.

ii. **Social-demographic information**

Interviewee ID………………… ………...	Interview location/district……………………….

Date of interview………………………	Sex of interviewee………………………………

Age of Interviewee…………………….	Profession of Interviewee……………………….

Education level………………………. Years of experience in a school-based PC …….…

**Section B: General understanding of program and Implementation Experiences**

1. Please tell how do you understand school-based preventive chemotherapy for schistosomiasis?
2. What are your roles and responsibilities in implementing school-based preventive chemotherapy for schistosomiasis?
3. Can you tell about capacity-building training you received to prepare you for this role?

**Section C: Barriers and facilitators influencing implementation of school-based PC**

i. **Barriers to Implementation Fidelity PCT for Schistosomiasis**
4. What are the barriers related to program coordination that hinder effective implementation?

**Probe**: How do these factors affect the implementation of the programs?

5. What barriers have you faced from the community during the implementation of this program?

Probe: How do these community factors affected effective implementation of the program?

ii. **Facilitators to Implementation Fidelity of PCT**

7. What factors do you perceive facilitated the effective implementation of this program?
8. Which strategies, if effectively implemented, can enhance the program?
9. How does a community support system facilitates implementation of this program?
10. Can you talk about supports you received from the government that facilitated your work?

**Section D: Adherence to school-based Preventive chemotherapy Protocol**

9. Can you tell me about school-based preventive chemotherapy implementation protocols?
10. Can you tell me how implementation of this program in this district adheres to the protocol?
11. Tell about the program coverage/do program reach all schools and all eligible children?
12. Can you tell about the content delivered (drug type, availability, and adherence to dosage)? and implementation time and program intervals/schedules?

**Section E: Determinants of adherence to preventive chemotherapy protocol**

13. In your opinion, which factors determine adherence to the protocols of this program?
14. Tell me how complex this program is to implement? And which aspects is too complex?
15. Tell about how parents, students and general community respond to this program?
16. Which strategies have been employed to increase adherence to the protocols

**Section F: Suggestions for Improvement**

13. What should be done to improve the implementation of this program in your district?

**Probe:** How would this change influence the outcomes of the program?

**Section G: Closing**

15. Is there anything else you would like to share about this program implementation?

***Thank you for your participation***
