# Supplementary material for: Contextual barriers and facilitators influencing implementation fidelity of school-based preventive chemotherapy for schistosomiasis: A qualitative study in two endemic districts in the Central Region, Ghana
Source: PLoS One. 2026 Aug 19;21(8):e0348920. doi: 10.1371/journal.pone.0348920 (PMC13489406; doi:10.1371/journal.pone.0348920)
Supplement: Supplementary file 7 [file pone.0348920.s007.docx]

**S2 Appendix: Key Informant Interview Guide.**

**Section A: Introduction and Demographic Overview**

i. **Introduction and rapport building**

Welcome to this short interview. The purpose of the study is to assess contextual barriers and facilitators influencing implementation fidelity of school-based preventive chemotherapy for schistosomiasis. I would like to ask you a few questions. Your information will be kept confidential, and you have the right to skip any question or withdraw at any time. Please allow me to proceed.

ii. **Social-demographic information**

Interviewee ID………………… ………...	Interview location/district……………………….

Date of interview………………………	Sex of interviewee………………………………

Age of Interviewee…………………….	Profession of Interviewee……………………….

Education level………………………. Years of experience in a school-based PC …….…

**Section B: General understanding of program and Implementation Experiences**

1. Please can you tell how implementation of school-based preventive chemotherapy for schistosomiasis is done in this district?
2. Which is your roles/responsibilities in implementation of this program?
3. Tell about capacity building training you received to prepare you to carry out these roles?

**Section C: Barrier and facilitators influencing implementation of school-based PC.**

i. **Barriers to Implementation fidelity PCT for Schistosomiasis**
4. What are the barriers related to program coordination that hinder effective implementation?

**Probe**: How do these factors affect implementation of the programs?

5. What barriers have you faced from community during coordination of this program?

Probe: How do this community factors affected effective implementation of the program?

ii. **Facilitators to Implementation Fidelity of PCT**

6. What factors do you perceive facilitated the effective implementation of this program?
7. Which strategies, if effectively implemented, can enhance success of the program?
8. Tell about teachers/health workers support that facilitated implementation of this program?
9. Which supports you received from GHS/GES that facilitated program implementation?

**Section D: Adherence to school-based Preventive chemotherapy Protocol**

9. Can you tell me about school-based preventive chemotherapy implementation protocol?
10. Can you tell me how implementation of this program in this district adheres to that protocol?
11. Tell about the program coverage/do program reach all schools and all eligible children?
12. Can you talk about the content delivered (drug type, availability, and adherence to dosage)? and implementation time and program intervals/schedules?

**Section E: Determinants of adherence to preventive chemotherapy protocol**

13. In your opinion, which factors determine adherence to the protocols of this program?
14. Tell me how complex this program is to implement? And which aspects is too complex?
15. Tell about how parents, students and general community respond to this program?
16. Which strategies have been employed to increase adherence to the protocol in your district?

**Section F: Suggestions for Improvement**

14. What should be done to improve the implementation of this program in your district?

**Probe:** How would this change influence the outcomes of the program?

**Section G: Closing**

16. What should be done to improve the implementation of this program in your district?

***Thank you for your participation***
