# Supplementary material for: Contextual barriers and facilitators influencing implementation fidelity of school-based preventive chemotherapy for schistosomiasis: A qualitative study in two endemic districts in the Central Region, Ghana
Source: PLoS One. 2026 Aug 19;21(8):e0348920. doi: 10.1371/journal.pone.0348920 (PMC13489406; doi:10.1371/journal.pone.0348920)
Supplement: Supplementary file 8 [file pone.0348920.s008.docx]

**S3 Appendix: Participant Information Sheet**

**Study title:** *Contextual Barriers and Facilitators Influencing Implementation Fidelity of School- Based Preventive Chemotherapy for Schistosomiasis in Gomoa East and Awutu Senya East Districts, Central Region, Ghana.*

**Principal Investigator**: Haruna Moshi

**Address**: Department of Social and Behavioral Science, School of Public Health.

P.O Box LG 13, University of Ghana, Legon.

TEL: 0206432963/Email: [harunamo2017@gmail.com](mailto:harunamo2017@gmail.com)/[hmoshi@st.ug.edu.gh](mailto:hmoshi@st.ug.edu.gh)

**General Information**

Hello! My name is Haruna Moshi, a Master of Public Health student at the University of Ghana. I am researching contextual barriers and facilitators influencing implementation fidelity of school-based preventive chemotherapy for schistosomiasis in Gomoa East and Awutu Senya East districts. I kindly invite you to participate in this study. Participation involves a brief interview, and your involvement would greatly contribute to improving school-based preventive chemotherapy program implementation. Thank you for considering this opportunity to make a positive impact.

**Risks and Discomforts**

There are no significant physical risks associated with participating in this study. However, you may feel uncomfortable discussing certain aspects of your beliefs or practices. If at any point you feel uncomfortable, you are free to skip any question or withdraw from the study at any time.

**Benefits**

While there are no direct personal benefits of participating in this study, your responses will help to improve the implementation of school-based preventive chemotherapy in your district.

The findings may help strengthen implementation strategies and policies and support better healthcare outcomes for school-aged children in Ghana.

**Confidentiality and anonymity**

Your privacy is very important. All responses will be kept strictly confidential. No identifying information will be collected. Data will be securely stored and accessible only to the research team. If results from this study are published, no personal identifying information will be included.

**Right to refuse or withdraw**

Please understand that your participation is voluntary. If you decide not to be part of this study, your decision will not affect your relationship with the staff of the Ghana Health Service in any way. You will also not lose any benefits that you would have otherwise been entitled.

**Further information**

This study has been reviewed and approved by the Ethical Review Committee of the Ghana Health Service. If you would like to find out more about the study or if you have any concerns, you may contact any of the following persons: Dr. Franklin N. Glozah (Academic Supervisor on 0572000534) or the Administrator of Ghana Health Service Ethics Review Committee on 0503539896 or Haruna Moshi (Principal Investigator on 0206432963. Are you willing to participate in the survey?	1. Yes [ ]	2. No [ ]

I have read the entire information given above and have agreed to participate in this study.

SIGNATURE OF PARTICIPANT: ........................................DATE……………………

NAME OF RESEARCHER:………………………………………………… SIGNATURE……………………………………………. DATE………………………
