# Supplementary material for: Contextual barriers and facilitators influencing implementation fidelity of school-based preventive chemotherapy for schistosomiasis: A qualitative study in two endemic districts in the Central Region, Ghana
Source: PLoS One. 2026 Aug 19;21(8):e0348920. doi: 10.1371/journal.pone.0348920 (PMC13489406; doi:10.1371/journal.pone.0348920)
Supplement: Supplementary file 9 [file pone.0348920.s009.docx]

**S4 Appendix: Informed Consent Form**

**TITLE:** *Contextual Barriers and Facilitators Influencing Implementation Fidelity of School- Based Preventive Chemotherapy for Schistosomiasis in Gomoa East and Awutu Senya East districts in the Central Region of Ghana.*

**INFORMED CONSENT FOR RESPONDENTS**

**Participant ID: / /_/……………….**

**PARTICIPANTS’ STATEMENT**

I ...........................................................................acknowledge that I have read or have heard the purpose and contents of the Participants’ Information Sheet, and all questions were satisfactorily explained to me in a language I understand. I fully understand the contents and any potential implications as well as my right to change my mind (i.e., withdraw from the research) even after I have signed this form.

I voluntarily agree to be part of this research.

Name of Participant/Respondent…………………………... Participants’/Respondents’Signature…………...or Thumb Print…………………………… Date………………………………

**Medium of communication:**

[ ] Read by self

[ ] Read and interpreted by interviewer/interpreter [ ] Consent through a witness

1. **Witness Details**
   I. ………………………was present when this study was explained to…………I can attest that this interviewee voluntarily agreed.to participate in the study.

Signature…………………………………. ………………. Date……………………………

2. **Details of interpreter**
   I. …………………… who voluntarily agreed to participate in the study, I read the consent form in (Ga/Ewe/Twi) and interpreted to……….,

Signature…………………………………. …Date……………………………

Investigator Statement and Signature

I certify that the participant/respondent has been given ample time to read and learn about the study. All questions and clarifications raised by the participants have been addressed.

Researcher’s name:…………………… Signature ……………………… Date……………
