# Supplementary material for: Contextual barriers and facilitators influencing implementation fidelity of school-based preventive chemotherapy for schistosomiasis: A qualitative study in two endemic districts in the Central Region, Ghana
Source: PLoS One. 2026 Aug 19;21(8):e0348920. doi: 10.1371/journal.pone.0348920 (PMC13489406; doi:10.1371/journal.pone.0348920)
Supplement: Supplementary file 12 [file pone.0348920.s012.docx]

# UNIVERSITY OF GHANA

**SCHOOL OF PUBLIC HEALTH
TDR POSTGRADUATE TRAINING SCHEME**

**Research Article Submission Approval Form**

This form must be completed by all TDR Scholarship students seeking approval to submit a manuscript derived from their dissertation research for publication. The information provided will help ensure compliance with institutional and TDR publication policies, authorship ethics, and acknowledgment of funding support.

## SECTION A: STUDENT INFORMATION

| Full Name | Haruna Moshi |
| --- | --- |
| Student ID Number | 22258040 |
| Programme (MPH/MSc) | MPH |
| Email Address | [harunamo2017@gmail.com](mailto:harunamo2017@gmail.com) |
| Mobile Phone Number | +255767753763 |
|  |  |

## SECTION B: MANUSCRIPT INFORMATION

| Proposed Title of Manuscript | Contextual Barriers and Facilitators Influencing Implementation Fidelity of School-Based Preventive Chemotherapy for Schistosomiasis: A qualitative study in Two Endemic Districts in the Central Region, Ghana. |
| --- | --- |
| Type of Article (Original Research / Review / Short Communication / Other | Original Research |
| Derived from Dissertation Title | Contextual Barriers and Facilitators Influencing Implementation Fidelity of School-Based Preventive Chemotherapy for Schistosomiasis in Gomoa East And Awutu Senya East Districts, Central Region, Ghana |
| Target Journal Name | PLOS ONE |
| Journal Category (Open Access / Subscription-based / Hybrid) | Open Access |
| Journal Impact Factor (if known) | 3.2 |
| Does the journal charge publication fees? (Yes/No) | Yes |
| If Yes, state amount (USD) | 2,382 |
| Has a waiver been requested? (Yes/No) | Yes |

## SECTION C: AUTHORSHIP DETAILS

List of Authors (in correct order):

| No. | Name (Surname, First) |
| --- | --- |
| First Author | Moshi, Haruna |
| Corresponding Author | Moshi, Haruna |

**Acknowledgment Statement (to appear in the manuscript):**

“This study was supported by the TDR Postgraduate Training Scheme at the School of Public Health, University of Ghana, funded by TDR, the Special Programme for Research and Training in Tropical Diseases, co-sponsored by UNICEF, UNDP, the World Bank, and WHO. The funders had no role in the study design, data collection, analysis, decision to publish, or preparation of the manuscript.”

## Has funding support been appropriately acknowledged in the manuscript?”

Yes	No

## SUPERVISOR CONFIRMATION

Dissertation Supervisor Name: Franklin N. Glozah

Approved:	Yes	No

Signature and Date: ____________	21^st^ April 2026

**COORDINATOR APPROVAL**

Approved:	Yes	No

Date: 21^st^ April 2026
