# Supplementary material for: Contextual barriers and facilitators influencing implementation fidelity of school-based preventive chemotherapy for schistosomiasis: A qualitative study in two endemic districts in the Central Region, Ghana
Source: PLoS One. 2026 Aug 19;21(8):e0348920. doi: 10.1371/journal.pone.0348920 (PMC13489406; doi:10.1371/journal.pone.0348920)
Supplement: Supplementary file 13 [file pone.0348920.s013.docx]

**A Codebook for Contextual Barriers and Facilitators Influencing Implementation Fidelity of School-Based Preventive Chemotherapy for Schistosomiasis: A Qualitative Study in Two Endemic Districts in the Central Region, Ghana.**

| A. **Barriers to Implementation Fidelity of School-Based Preventive Chemotherapy for Schistosomiasis** | | | | | | |
| --- | --- | --- | --- | --- | --- | --- |
|  | **Theme** | **Subtheme** | **Definition** | **Excerpts** | **CIFR Domain** | **Fidelity Component** |
|  | Socio-cultural beliefs and misconceptions on treatment uptake | Perceptions and myths surrounding school-based preventive chemotherapy | Misconceptions that praziquantel causes infertility, poisoning, or hidden population-control intentions. | *“The big problem here is that people think the drugs we give out for free are poison and cause infertility… These are super drugs, and they have something to do with controlling birth.... That is their perception.” (KII, NTD Program Coordinator 1)*  *“The moment they hear 'mass medication,' suspicions rise. Some think it’s to control their children or secretly test drugs.” (IDI, SHEP teacher 5)*  *“In the market, I overheard someone say, ‘This is why we don’t take government drugs today, it's worms, tomorrow it's infertility.’” (IDI, SHEP teacher 4* | Outer Setting | Coverage |
|  |  | Parental resistance to school-based preventive chemotherapy | Parents discourage or prevent children from participating in MDA activities. | *“You see parents picking their kids up early, some telling me not to give my child anything..., Some don’t come to school at all on the day of administration because their parents tell them not to go. It's becoming a trend.” (IDI, Head Teacher 2).*  *“Parents came to school and warned us not to give any medication to their children. They said we’ll be responsible if anything happens. It puts us in a tough position.” (IDI, SHEP teacher 2)* | Outer Setting | Coverage |
|  |  | Cultural and religious beliefs | Religious teachings and cultural norms discourage treatment uptake. | *“I remember one pastor advised students not to take any medication given in schools, he called it ‘Satan’s strategy to weaken children.’” (IDI, Head Teacher 1)*  *“During fasting, some Muslim students said taking the drugs would break their fast, and they refused.” (IDI, SHEP teacher 7).* | Outer  Setting | Participant Responsiveness |
|  | Drug-related fears and adverse reactions | Actual adverse reactions to praziquantel | Experiences of dizziness, vomiting, abdominal pain, fainting, and menstrual-related concerns following treatment. | *“One girl collapsed and vomited after taking the medication. We later learned she hadn’t eaten since morning. It’s common, especially in schools without a feeding program.” (IDI, Head Teacher 4)*  *“Some students bled more than usual during menstruation after taking the drug, and that scared parents.” (IDI, Head teacher 1)*  *“They react because the tablets are strong and big, and if there’s no food in the stomach, it hits harder.” (IDI, SHEP teacher 8)* | Inner Setting | Quality of Delivery |
|  |  | Fear of side effects and psychosomatic reactions | Fear and anxiety about treatment trigger refusal and psychosomatic symptoms | *“I went to one school and found about 50 students lying down claiming they felt dizzy, yet they had not even taken the medication. It was all fear.” (KII, NTD Program Coordinator 1)*  *“As soon as the first child said she felt faint, others followed. It’s like a chain reaction based on panic.” (IDI, SHEP teacher 4)*  *“They get scared, especially when classmates pretend to faint or cry. It spreads quickly.” (IDI, Head teacher 6)* | Characteristics of Individuals | Participant Responsiveness |
|  | Logistical constraints and resource limitations | Inadequacy of measuring tools | Insufficient dose poles and measuring tapes compromise accurate dosage determination. | *“We had to draw height charts on the classroom wall using chalk and estimate dosage by comparison. It’s not ideal, but what choice did we have?” (IDI, SHEP Teacher 4)*  *“Our district got just 80 measuring tapes for over 300 schools. We shared among clusters and improvised the rest.” (KII, NTD program coordinator 1)*  *“I used a string and marked it for different heights based on last year’s tape. It’s not accurate but better than nothing.” (IDI, SHEP teacher 2)* | Inner Setting | Quality of Delivery |
|  |  | Transportation challenges | Limited transport support for drug distribution and supervision activities. | *“When we couldn’t hire a vehicle, I used a motorbike for nearly 40 km. That’s not safe or sustainable.” (IDI, SHEP Teacher 5)*  *“No one thinks about how the medicines actually get to rural schools; we pay out of pocket or borrow a ride.” (IDI, SHEP teacher 9)* | Inner Settings | Facilitation Strategy |
|  | Lack of training and incentives for frontline implementers | Inadequate teachers training | Limited training opportunities reduce teachers’ knowledge and confidence in implementation. | *“Only one teacher per school is invited to the training. When that person is absent or uninterested, the rest are left guessing on what to do”* (KII, NTD Program Coordinator 1)  *“Training should include at least two or three staff. It’s risky when one person holds all the knowledge.”* (IDI, Head teacher 3) | Inner Setting | Quality of Delivery |
|  |  | Lack of incentives for implementers | Absence of financial and non-financial motivation affects commitment and participation. | *“Teachers are expected to do a lot of extra work during MDA, but there is no motivation attached to it.”* (IDI, SHEP Teacher 6) | Characteristics of an individual | Quality of delivery |
|  | Inadequate community sensitization and engagement | Limited pre-MDA community sensitization | Insufficient awareness activities and Inadequate involvement of parents and community leaders contribute to misconceptions and resistance. | *“Funds are released late, and there’s barely time to conduct sensitization. We end up rushing the process, and parents aren’t properly informed.”* (KII, NTD Program Coordinator *2)*  *“Some communities hear about the drug distribution only on the morning of the event. That’s not enough to build trust.” (IDI, SHEP Teacher 8)* | Process | Quality of delivery |
|  | Insufficient program funds | Inadequate program funding or a delay in fund release. | Lack of funds and Late disbursement of funds affect implementation planning and execution. | *“The biggest challenge is delayed funding. By the time funds arrive, there is very little time left for community sensitization and preparation.” (KII, NTD Program Coordinator 2)*  *“Sometimes we skip MDA for several years because we don’t have money to implement it…, the last time we conducted it was 2021” (IDI, SHEP Teacher 9).*  *“There’s always a late release of funds…, training and all that stuff is usually done a few days before the program in a rush.” (KII, NTD Program Coordinator 1).* | Inner setting | Adharance |
|  | Gaps in Coverage and Fidelity to Protocol | Low program coverage | The inability of the preventive chemotherapy programme to reach all eligible school-aged children due to absenteeism, parental refusal, exclusion of out-of-school children, and other implementation challenges results in lower treatment coverage than intended. | *“We are never at 100 percent in reaching schools and students…, Coverage is always low; we have several schools that refuse to participate, especially the private schools.…, Some were not registered in the district, others are new schools that have not been incorporated into the program”. (KII, NTD Program Coordinator 1).*  *“Many children miss treatment because they are absent on the day the drugs are administered.” (IDI, Head Teacher 3)*  *“Out of school children hardly come for the drug, maybe 50 out of 100,000 children; some children do not attend on a day of MDA, and some who attend were not able to take the drug because they reject… So generally, we have low coverage.” (IDI, SHEP coordinator 2).* | Implementation Process | Coverage |
|  |  | Short implementation duration | Insufficient time allocated for planning, community sensitization, training, drug distribution, and follow-up activities, leading to rushed implementation and reduced adherence to programme protocols. | *"We do the exercise for only one day…if the child is absent, then he/she simply misses it out. There’s no follow-up, no make-up day.” (IDI, SHEP Teacher 2).*  *“The time is too short, only a day in each school. The ones not there are missing out, and that’s how you get lower coverage.” (KII, NTD Program Coordinator 4)* | Process | Adherence |
|  |  | Inconsistent program frequency | Irregular implementation of preventive chemotherapy campaigns and deviations from the recommended treatment schedule reduce programme continuity, community familiarity, and sustained disease control efforts. | *“The last time we did it was three years ago… there has not been stability. This program has no constant schedule, children miss treatments.” (KII, NTD Program Coordinator 1).*  *“Skipping years diminishes an impact… transmission of schistosomiasis goes on, because these children are not treated all the time” (KII, NTD Program Coordinator 4).*  *“If it is every year, parents and kids would get to know and prepare, but now it is coming after three years or more, for instance, the last MDA was in 2021, so when it comes again, resistance will be even more (IDI, Head Teacher 5).* | Process | Frequency (Dose) |
| B. **Facilitators of Implementation Fidelity of School-Based Preventive Chemotherapy for Schistosomiasis** | | | | | | |
|  | **Theme** | **Subtheme** | **Description** | **Excerpts** | **CIFR Domain** | **Fidelity Component** |
|  | Commitment of frontline implementers and role modelling | Teachers’ commitment to students’ health | Teachers demonstrate ownership and commitment to successful implementation. | *“We see it as part of our responsibility to protect the health of our students.”* (IDI, SHEP Teacher 7)  *When it comes to deworming, I stop other tasks and ensure all students take the drugs. I really care about students’ health…, because I’m a victim of schisto”.* (IDI, Head Teacher 1).  *“We explain what schisto is, what worms are, and why we need the drugs. Once students understand, they cooperate better.” (IDI, SHEP teacher 8)*  *“I use diagrams and show urine samples so students know why it matters.”* (IDI, SHEP teacher 2) | Inner Setting | Adherence |
|  |  | Teachers’ role modelling of swallowing drugs | Teachers take praziquantel in front of students to reassure them. | *“I always take mine first. The children watch me…, When the student sees that their teacher doesn’t suffer any side effects, they all want to be first in line for his tablets. It makes a difference.” (IDI, SHEP Teacher 8).*  *“Some teachers even take videos or explain how it feels after swallowing, just to reduce fear.” (IDI, Head teacher 1)*  *“When students see that nothing happens to their teachers, they start lining up to receive the tablets.” (KII, District SHEP Coordinator 2)* | Characteristics of Individuals | Participant responsiveness |
|  | Pre-MDA sensitization/awareness | Pre-MDA Parent-Teacher Association (PTA) meetings | PTA meetings are used to educate parents about the program before implementation. | *“We use PTA meetings to explain the purpose of the exercise and answer parents’ concerns*.” (IDI, Head Teacher 2)  *“Mmh…, yes, we do explain the MDA program through PTA Meetings, we call the parents, that ‘we are going to start deworming your children, it is safe…. It works very much”* (IDI, Head Teacher 1). | Process | Covarage |
|  |  | Community mobilization before MDA. | Awareness and education activities increase community understanding and acceptance. | *When we take time to educate the community before implementation, acceptance improves significantly.”* (KII, NTD Program Coordinator 2)  *“We do explain to the parents and students before we give drugs…, we assure them that it's safe. We move out with a microphone to announce* (IDI, SHEP Teacher 9).  *We explain what schisto is, what worms are, and why we need the drugs. Once students understand, they cooperate better.”* (IDI, SHEP Teacher 8) | Process | Covarage |
|  |  | Engagement of the community in the program | Engagement of the local community and leaders increases ownership, confidence, and participation in the program. | *“We sometimes engage local leaders before we give drugs…, We move out with them to assure the community it's safe* (IDI, SHEP Teacher 4). | Process | Covarage |
|  | **Collaboration between the health and education sectors** | Joint implementation between GHS and GES. | Joint implementation and oversight by GHS and GES improve coordination and implementation efficiency. | *“The health and education sectors work together throughout the implementation process.”* (KII, District SHEP Coordinator 1)  *“We plan together and solve challenges together.”* (KII, District SHEP Coordinator 2)  *“Last year, our school got full support from GES and GHS teams.” (IDI, Head teacher 4)* | Process | Quality of delivery |
|  |  | Supportive supervision for teachers | Continuous technical guidance, mentorship, monitoring, and feedback provided by district health and education officials to teachers during planning and implementation improved the quality and consistency of preventive chemotherapy delivery. | *“They sometimes call after the drug day to confirm if we followed protocol. That keeps us alert.”* (IDI, SHEP Teacher 5)  *“Anytime they come here to us from the district or regional headquarters, they do supervision and assist us in technical aspects. They ensure we get everything done, and we are correct in our documentation.”* (IDI, SHEP teacher 9).  *“Sometimes nurses visit us for help to execute programs. When they come around, they guide us and solve any issue immediately.”* (IDI, SHEP Teacher 6). | Process | Quality of Delivery |
|  |  |  |  |  |  |  |
| C. **Recommended Strategies for Improving Implementation Fidelity** | | | | | | |
|  | **Theme** | **Subtheme** | **Description** | **Excerpts** | **CIFR Domain** | **Fidelity Component** |
|  | **Integrated Drug Delivery Approaches** | Complementing school-based MDA with community-based distribution | Community-based delivery improves access for children who missed treatment during the school-based MDA campaign. | *“Many children miss treatment because they are absent from school or not enrolled. Community-based distribution can help ensure that these children are also reached.”* (NTD Program Coordinator 4)  *“The school approach is effective, but it cannot reach every child. If community health workers are involved in distribution at the community level, more children will benefit from the program.*” (SHEP Coordinator 2) | Outer settings |  |
|  |  | Expanding coverage to out-of-school children | Deliberate strategies to reach non-enrolled children at their homes and at healthcare facilities. | *“The program should include mechanisms for reaching children who are not attending school, either by visiting their homes or even when they attend health facilities.”* (District SHEP Coordinator 1) |  |  |
|  |  | Strengthening school-based outreach activities | Follow-up outreach to reach missed children | *“Schools should be supported to conduct follow-up outreach for children who miss treatment days.”* (Head Teacher 7)  *“Some children are absent on the day of drug administration and never receive treatment. Follow-up outreach activities could help ensure that these children are not left behind.”* (Head Teacher 3) |  |  |
|  | **Technology-Assisted Monitoring and Reporting Systems** | Mobile-based reporting applications | Digital reporting tools to improve reporting and monitoring of distribution activities. | *“Digital reporting systems could help us monitor implementation more effectively and identify coverage gaps early enough for corrective action.”* (NTD Program Coordinator 3)  *“If teachers could submit reports through mobile applications, it would reduce delays and make it easier to track implementation progress from the district level.”* (NTD Program Coordinator 2) | Implementation Process |  |
|  |  | Digital dashboards and electronic tracking systems | Real-time monitoring of implementation progress and coverage. | *“Electronic systems would allow us to track implementation progress and quickly identify areas needing attention.”* (District SHEP Coordinator 2) |  |  |
|  |  | GIS-based verification of treatment coverage | Geographic tracking and verification of treatment coverage. | *“GIS technology could help verify whether all targeted communities and schools have been reached.”* (NTD Program Coordinator 4)  *“Sometimes it is difficult to know whether all targeted schools and communities have been reached. A digital tracking system and GIS systems could help verify coverage and support better supervision.” (SHEP Coordinator 1)* |  |  |
|  | **Improved Praziquantel Formulations** | Development of child-friendly formulations with a smaller tablet size | There should be development of new, smaller tablets that are easy to swallow and improve administration and acceptance. | *“Some children struggle with the size of the tablets and become afraid after seeing others experience side effects. Smaller tablets with fewer side effects would make administration easier.”* (SHEP Teacher 7)  *“Many children are frightened when they see the tablets because they are large and difficult to swallow. If the medicine was smaller and easier to take, acceptance would be much better.”* (Head Teacher 4) | Characteristics of intervention |  |
|  |  | Reduced side effects and improved palatability | The new praziquantel formulation, which has no or few side effects and tastes better, will improve acceptability. | *“When children experience side effects or hear about them from their friends, it affects participation during subsequent campaigns. Formulations that are better tolerated could improve confidence in the program.”* (NTD Program Coordinator 1)  *“If the medicine causes fewer unpleasant reactions, more children will be willing to take it.”* (NTD Program Coordinator 2) |  |  |
|  | **Enhanced Community Sensitization and Stakeholder Engagement** | Early and continuous community sensitization | Sensitization should start earlier and continue throughout | *“Sensitization should not be done a few days before the exercise. It should start early and involve parents so they understand why the children need the medicine.” (SHEP Teacher 4)*  *“Most of the refusals we encounter come from misinformation within communities. If parents and community leaders receive accurate information before the exercise, acceptance of the drugs will improve significantly.” (NTD Program Coordinator 2)* | Implementation Process |  |
|  |  | Strengthening parental engagement through PTA meetings | Increased parental involvement before implementation. | *“If parents receive accurate information before the exercise, acceptance of the drugs will improve significantly.” (NTD Program Coordinator 2)* |  |  |
|  |  | Involvement of community, religious, and traditional leaders | Trusted community actors should participate in sensitization efforts. | *“Pastors, imams, chiefs, and other community leaders should be well involved in educating the community; people are more likely to trust their information and allow their children to participate in the program”. (Head Teacher 2)*  *“People in the community listen to their pastors, imams, and traditional leaders. If these leaders are involved in educating the community, it will help reduce misconceptions and improve acceptance of the drugs.” (SHEP Teacher 6)* |  |  |
