# Supplementary material for: Contextual barriers and facilitators influencing implementation fidelity of school-based preventive chemotherapy for schistosomiasis: A qualitative study in two endemic districts in the Central Region, Ghana
Source: PLoS One. 2026 Aug 19;21(8):e0348920. doi: 10.1371/journal.pone.0348920 (PMC13489406; doi:10.1371/journal.pone.0348920)
Supplement: Supplementary file 16 [file pone.0348920.s017.docx]

**Response to the Academic Editors' and Reviewers' Comments for Manuscript ID: PONE-D-26-20044 “Contextual Barriers and Facilitators Influencing Implementation Fidelity of School-Based Preventive Chemotherapy for Schistosomiasis: A Qualitative Study in Two Endemic Districts in the Central Region, Ghana”**

We sincerely thank the Academic Editor and Reviewers for their constructive comments and suggestions. We have carefully revised the manuscript and addressed all comments as detailed below.

| **PART A: RESPONSE TO ACADEMIC EDITORS’ COMMENTS** | | |
| --- | --- | --- |
| **Comment** | **Response** | **Location** |
| **Academic Editor:** Please ensure that your manuscript meets PLOS ONE style requirements. | We have carefully revised the manuscript to ensure compliance with all PLOS ONE formatting and style requirements, including manuscript structure, references, tables, figures, and supporting files. | Throughout manuscript |
| **Academic Editor:** Please include the PLOS questionnaire on inclusivity in global research. | We have completed the Inclusivity in Global Research Questionnaire and uploaded it as Supporting Information as requested. | Supporting Information |
| **Academic Editor:** Please clarify the role of the funders. | The funding statement has been revised to clarify the role of the funders. We have added the statement: ***“The funders had no role in study design, data collection and analysis, decision to publish, or preparation of the manuscript.”*** | Funding Statement in Manuscript Submission Portal |
| **Academic Editor:** Remove funding information from the manuscript text. | All funding-related information has been removed from the manuscript body and retained only within the Funding Statement section of the manuscript submission portal. | Funding Statement in Manuscript Submission Portal |
| **Academic Editor:** Revise Data Availability Statement to provide a non-author institutional contact. | The Data Availability Statement has been revised to include a non-author institutional contact point in accordance with PLOS ONE data sharing requirements. | Data Availability Statement in the manuscript submission portal |
| **Academic Editor:** Supporting Figure 2 contains potentially copyrighted map images. | The copyrighted map has been totally removed from the manuscript and no longer exists as a supporting Figure 2. | Supporting Information |
| **Academic Editor:** Upload Supporting Information Tables referred to in the manuscript | All supporting tables cited in the manuscript have now been uploaded and appropriately referenced. | Supporting Information (Table 1-4) |
| **PART B: RESPONSE TO REVIEWERS’ COMMENTS** | | |
| **Reviewer #1** | | |
| **Comment** | **Response** | **Location** |
| Scientific names should be in italics (*Schistosoma mansoni* and *Schistosoma haematobium*). | All scientific names have been italicized throughout the manuscript. | Page 1, Lines 62-64 |
| Lines 73-80 contain long sentences; split them. | The identified sentences have been revised and divided into shorter sentences to improve readability and clarity. | Page 1, Lines 73-80 |
| Standardize in-text citations and references to meet PLOS ONE journal format (Kwabi et al., 2024), (Damschroder et al., 2022), and (Carroll et al., 2007). | All references and in-text citations have been standardized according to PLOS ONE citation style. | Throughout manuscript |
| Table 1 should include the sample size (N) in its column and remove “Years” since that is already included in the table header. | Table 1 has been revised to include sample size (N), and the redundant “Years” has been removed. | Table 1, Page 9-10, line 244 |
| Inclusion and exclusion criteria are not clearly defined. | The inclusion and exclusion criteria have been expanded and clearly described in the Methods section. | Page 5, Lines 155-160 |
| Please define implementation fidelity. | A definition of implementation fidelity has been added to the Introduction section with an appropriate citation. | Page 3, Lines 114-116 |
| Ethical considerations: What language was the information sheet written in? How were non-literate participants handled? | The Ethical Considerations section has been expanded to explain that participant information sheets and consent forms were provided in English and explained in participants’ preferred language when necessary. Procedures for obtaining informed consent from participants unable to read or write have also been described. | Page 7-8, Lines 202-216 |
| Explain triangulation procedures. | A detailed description of triangulation procedures has been added. We clarified how data from different participant groups and districts were compared to enhance credibility and trustworthiness. | Page 8, Lines 220-224 |
| Lines 205–209 contain a very long sentence. | The sentence has been revised and divided into shorter sentences for improved clarity. | Page 7, Lines 205-209 |
| Gender balance (11 women and 10 men): Was this intentional? | We have clarified that a nearly gender balance was not intentionally targeted during recruitment. Rather, participant selection was based on eligibility criteria and involvement in program implementation activities. | Page 5, Lines 155-160 |
| Participant distribution across districts should be in Methods rather than Results. | The statement describing participant distribution across districts has been moved from the Results section to the Methods section. | Page 5, Lines 151 |
| Framework components are not evident in the Results and Discussion. | The Results and Discussion sections have been substantially revised to explicitly align findings with the CFIR domains (Outer Setting, Inner Setting, Characteristics of Individuals, and Process) and implementation fidelity framework constructs. | Results and Discussion Sections, including in S2, S3, and S4 tables |
| Use a qualitative reporting guideline. | The manuscript was reviewed against the COREQ checklist to strengthen reporting transparency and completeness. The completed checklist has been included as Supporting Information. | Supporting Information |
| Please provide the codebook. | The qualitative codebook has been developed and uploaded as Supporting Information (S1 Codebook). | Supporting Information (S1 Codebook) |
| **Reviewer #2** | | |
| **Comment** | **Response** | **Location** |
| Sentence on lines 163-165 is hanging. | The sentence has been revised for completeness and clarity. | Page 5, Lines 163-165 |
| Section 4 of the data collection tools indicates that you have collected information on recommended strategies for enhancing implementation fidelity. However, the result is not presented in the results section. Kindly present the results from the various study participants on the theme. | A new theme titled “Recommended Strategies for Enhancing Implementation Fidelity” has been added to the Results section. This section presents recommended strategies for enhancing implementation fidelity from participants’ perspectives. | Page 26-31, Lines 539-645 |
| **Reviewer #3** | | |
| **Comment** | **Response** | **Location** |
| You mean schools that do not offer a feeding program? Why is it in brackets? | The statement has been revised for clarity, and the brackets have been removed. The text now clearly refers to schools without feeding programs. | Page 14, Lines 306-307 |
| Why is eating before taking praziquantel not always possible in the districts? | Contextual explanation has been provided regarding household economic challenges and the absence of school feeding programs in some schools. | Page 14, Lines 308-310 |
| NTD Program Coordinator 1 shared many experiences compared to others. Is there a reason? | Additional quotations from other participants have been incorporated where appropriate to ensure balanced representation of perspectives across respondents. | Results Section |
| Who is responsible for releasing funds? | The manuscript has been revised to clarify the administrative structures and funding pathways responsible for releasing implementation funds. | Page 19, Lines 421-424 |
| What does 1.5 refer to? | The value has been clarified within the text to remove ambiguity. | Page 18, Line 401 |
| Was Coordinator 3 not interviewed? Her views are not included. | Coordinator 3 was interviewed. Quotations from this participant have been incorporated where relevant. Where views overlapped substantially with those of other participants, findings were synthesized to avoid repetition. | Page 19, Lines 410-412, Page 27, lines 557-559, and Page 28, lines 583-585 |
| Recast sentences on lines 582–584. | The sentences have been revised and simplified for clarity. | Page Lines 582-584 |
| Explain the meaning of temporal and structural dimensions. | The terminology has been clarified and replaced with more accessible language describing the timing of implementation activities and program delivery arrangements. | Page 35. Line 728-732 |
| Repetition in lines 597 and 602. | Repetitive wording has been removed and the paragraphs revised accordingly. | Page 36, Lines 745-747 |
| Elaborate on temporal consistency. | Additional explanation has been provided to clarify that temporal consistency refers to the regularity, predictability, and continuity of implementation activities over time. | Page 37, Lines 743-745 |

We sincerely thank the Academic Editor and Reviewers for their comments and suggestions. We believe that these responses have addressed all reviewers’ comments accordingly and have improved the clarity, rigor, and contribution of our study to implementation science and neglected tropical disease control.
